# SIRT7: an influence factor in healthy aging and the development of age-dependent myeloid stem-cell disorders

**DOI:** 10.1038/s41375-020-0803-3

**Published:** 2020-03-25

**Authors:** Alexander Kaiser, Martin Schmidt, Otmar Huber, Jochen J. Frietsch, Sebastian Scholl, Florian H. Heidel, Andreas Hochhaus, Jörg P. Müller, Thomas Ernst

**Affiliations:** 1 Klinik für Innere Medizin II, Abteilung Hämatologie und Internistische Onkologie, Universitätsklinikum Jena, Jena, Germany; 2 Institut für Biochemie II, Universitätsklinikum Jena, Friedrich-Schiller-Universität, Jena, Germany; 3 Leibniz-Institute on Aging (Fritz-Lipmann-Institute), Jena, Germany; 4 Institut für Molekulare Zellbiologie, CMB, Universitätsklinikum Jena, Friedrich-Schiller-Universität, Jena, Germany

**Keywords:** Leukaemia, Cancer stem cells

## Abstract

Molecular alterations within the hematopoietic system influence cellular longevity and development of age-related myeloid stem-cell disorders like acute myeloid leukemia (AML) and chronic myeloid leukemia (CML). A reduced SIRT7-expression in aged murine hematopoietic stem cells (HSC) resulted in reduced longevity and increased proliferation. In this study we investigated age-related changes of SIRT7-expression in healthy humans and relevant pathomechanisms in AML and CML. SIRT7-expression in leukocytes of healthy people decreased in an age-dependent manner. Low SIRT7 mRNA levels were also detected in AML and CML patients. With positive treatment response, SIRT7-expression increased, but showed reduction when patients progressed or relapsed. Pharmacologic inhibition of driver mutations in AML (FLT3-ITD) or CML (BCR-ABL) also restored SIRT7 levels in cell lines and patient samples. Furthermore, SIRT7-expression increased with time during PMA-mediated monocyte differentiation of THP-1 cells. SIRT7-overexpression in THP-1 cells resulted in increased expression of differentiation markers. BCR-ABL, FLT3-ITD, and differentiation-associated SIRT7-expression in general were positively regulated by C/EBPα, -β, and -ε binding to two different C/EBP-binding sites within the SIRT7 promoter. SIRT7 is important in human hematopoietic cell aging and longevity. It might act as tumor suppressor and could potentially serve as general biomarker for monitoring treatment response in myeloid stem-cell disorders.

## Introduction

Alterations within the hematopoietic system are a normal physiological consequence of the healthy aging process [1]. However, sometimes changes have a pathophysiological impact [2], for example, in the development of age-related leukemias, such as acute myeloid leukemia (AML) and chronic myeloid leukemia (CML). Though detailed molecular mechanisms of the aging hematopoietic system and dysfunctional hematopoiesis are often unknown, physiological, and pathophysiological mechanisms can be very closely associated. In this context, hematopoietic stem cell (HSC) changes in relation to longevity is a major focus of current research interests, not only because these cells have the potential to become leukemic as a result of genetic alterations, but also because progenitors may reacquire stem-cell characteristics such as self-renewal in the development of leukemic disease. Known hallmarks of aging include epigenetic alterations and mitochondrial dysfunction. Both changes can be influenced by deacetylation processes.

Sirtuins are a family of NAD^+^-dependent protein deacetylases. Seven sirtuins were described in mammals which are associated to many cellular activities, including energy metabolism, stress resistance, maintenance of genomic stability, aging, and tumorigenesis. Since histone acetylation undergoes dynamic changes during differentiation, the sirtuins have been linked to the development and differentiation of embryonic or HSCs [3]. Recently, SIRT2 was established as SIRT2 required for HSC maintenance and regenerative capacity at an old age by repressing the activation of the NLRP3 inflammasome in HSCs cell autonomously [4]. Mohrin et al. described the interaction of the class III histone deacetylase sirtuin 7 (SIRT7) with nuclear respiratory factor 1 (NRF1) in murine HSCs [5–7]. High SIRT7 levels were typically found in young adult HSCs. They inhibited NRF1-mediated transcription initiation of mitochondrial ribosomal proteins. As a result, mitochondrial biogenesis and energy metabolism were low, and HSCs remained quiescent ensuring HSC longevity. Conversely, older HSCs had low SIRT7 levels. NRF1 was active and mitochondrial biogenesis and energy metabolism were increased. This resulted in enhanced cell proliferation—a hallmark of cancer development [8]. In addition to the results of Mohrins et al., Vazquez et al. described accelerated aging and genome instability in SIRT7 knockout mice [9]. Next to the described role in aging and proliferation with tumorigenesis, it plays an important role in ribosome biogenesis, general transcription regulation, metabolic homeostasis, stress response, and genome stability [10]. The first characterization of the SIRT7 gene was performed by Voelter-Mahlknecht et al. [11], who localized the gene on chromosome 17q25.3—a region frequently altered in acute leukemias and lymphomas. The genomic sequence has a length of 6.2 kb with ten exons and one alternative exon 3a. The flanking promoter region is TATA- and CCAAT-boxless and lacks CpG islands. Ensembl Genome Browser searches revealed 21 splice variants in transcription products (ENSG00000187531) with only two protein-coding variants (SIRT7-210 and SIRT7-201). The UniProtKB database indicates three protein-coding isoforms (Q9NRC8; isoform 1 44.9 kDa, isoform 2 20.4 kDa, and isoform 3 35.9 kDa). Detailed information about principles of promoter regulation, potential exon-skipping mechanisms, posttranscriptional or posttranslational modifications, and processing are currently unknown.

The following study aimed to investigate the age dependency of intracellular SIRT7 levels in human hematopoietic cells and the association of low SIRT7 levels to the age-dependent malignant myeloid stem-cell disorders AML and CML. Moreover, the regulation of the SIRT7 promoter by transcription factors CCAAT-enhancer-binding proteins (C/EBP) α, β, and ε, and the effects on the functions and pathomechanisms mediated by SIRT7 were investigated.

### Patients and methods

All chemicals used were of analytical or cell culture grades. All oligonucleotides were purchased from Eurofins Genomics (Ebersberg, Germany).

### Preparation of patient samples

Peripheral blood or bone marrow samples were obtained from healthy donors (*n* = 169), CML (*n* = 78), or AML (*n* = 113) patients and collected in EDTA-tubes. Leukocytes were isolated after erythrocyte lysis according to standard protocols. RNA extraction of leukocytes and cell lines was performed using TRIzol reagent (Invitrogen, Carlsbad, USA) as previously described [12]. Complementary DNA was synthesized with the High-Capacity cDNA Archive Kit (Applied Biosystems, Foster City, USA) using random hexameric primers. This study was approved by the Jena University Hospital ethics committee (No. 3944-12/12).

### Quantitative real-time PCR (qRT-PCR)

SYBR green‐based analyses were performed using the Mastercycler® ep realplex Real‐time PCR System (Eppendorf, Hamburg, Germany). Primers and conditions are described in Supplementary Table 1. Housekeeper-gene in human cells or cell lines was β-glucuronidase. In murine cells β-actin was used.

### Exon-specific PCR: analysis of expressed SIRT7-exons

Transcription of all putative exons of the human SIRT7 gene was analyzed by reverse transcription PCR (RT-PCR) as described in Supplementary Fig. 1.

### Cells and cell culture

Different CML- and AML-cell lines were used as described in Supplementary Table 2. Intracellular processes were inhibited by FLT3-ITD inhibitor quizartinib (AC220; 20 nM), BCR-ABL inhibitors nilotinib (20–320 nM), imatinib (2000 nM), and dasatinib (10 nM) (all Cayman Chemical, Ann Arbor, USA). THP-1 monocyte cell differentiation was carried out by 5 days phorbol 12-myristate 13-acetate (PMA, 5 ng/ml, Cayman Chemical) incubation according to the protocol of Tsuchiya et al. [13]. For cytomorphological examination cells were Pappenheim stained [14] and visualized using an Axio Scope.A1 microscope (Zeiss, Jena, Germany).

### SIRT7-overexpression: production of pseudoviral particles and cell transduction

For the production of retroviral particles plasmids encoding SIRT7 WT (pBABE-SIRT7 WT) or enzymatically inactive SIRT7 (pBABE-SIRT7 H_187_Y) was used [15, 16]. Details are described in the supplementary information.

### Western blot analysis

Western blot analyses were done as described by Kyhse-Andersen [17]. Details and antibodies are described in the supplementary information (antibodies see Supplementary Table 3).

### Quantification of cell surface molecules by flow cytometry (FACS)

Measurements were done using the FACSCalibur analyzer and Cell QuestTM software (BD Bioscience, Heidelberg, Germany) according to the manufactures protocol (antibodies see Supplementary Table 3).

### Plasmids

All used plasmids are described in Supplementary Table 4. Furthermore, generation of hC/EBPα expression plasmids and SIRT7-promoter reporter-gene plasmids are explained in the supplementary information (Supplementary Fig. 2 and Supplementary Table 4).

### Transfection and luciferase reporter-gene assays

Ba/F3 cells and AR230 cells were transfected using the Nucleofector^TM^ I instrument with the Cell Line Nucleofector® Kit V (Lonza, Basal, Switzerland) according to manufacturer’s protocol using the program X-001. To quantify C/EBP-dependent promoter activities, the firefly luciferase-containing pGL3-promoter SIRT7 plasmid and Renilla luciferase-containing pRL-SV40 plasmid were used. Furthermore, different C/EBP-overexpression plasmids were co-transfected (Supplementary Table 4). Reporter-gene assay measurements were done as described previously [18].

### Chromatin immunoprecipitation (ChIP)

For in vivo evaluation of SIRT7-promoter binding by C/EBPs the SimpleChIP^®^ Enzymatic Chromatin IP Kit with magnetic beads (Cell Signaling Technology, Danvers, USA) was used according to manufacturer’s instructions. Antibodies for immunoprecipitation are described in Supplementary Table 3. Quantification of C/EBP-bound DNA was performed in a StepOnePlus™ Real-Time PCR system (Applied Biosystems) using primers and conditions summarized in Supplementary Table 1.

### Survival analysis

Survival in SIRT7 high and SIRT7 low-expressing patient groups was analyzed in FLT3-ITD-mutated and FLT3-wild-type AML patients. Detailed information is given in the supplementary information.

### Statistics

Statistical analyses and generation of figures were carried out using the SigmaPlot 13 software. Detailed information is given in the supplementary information and figures.

## Results

### SIRT7 gene expression in leukocytes of healthy people is age dependent

**Fig. 1:**
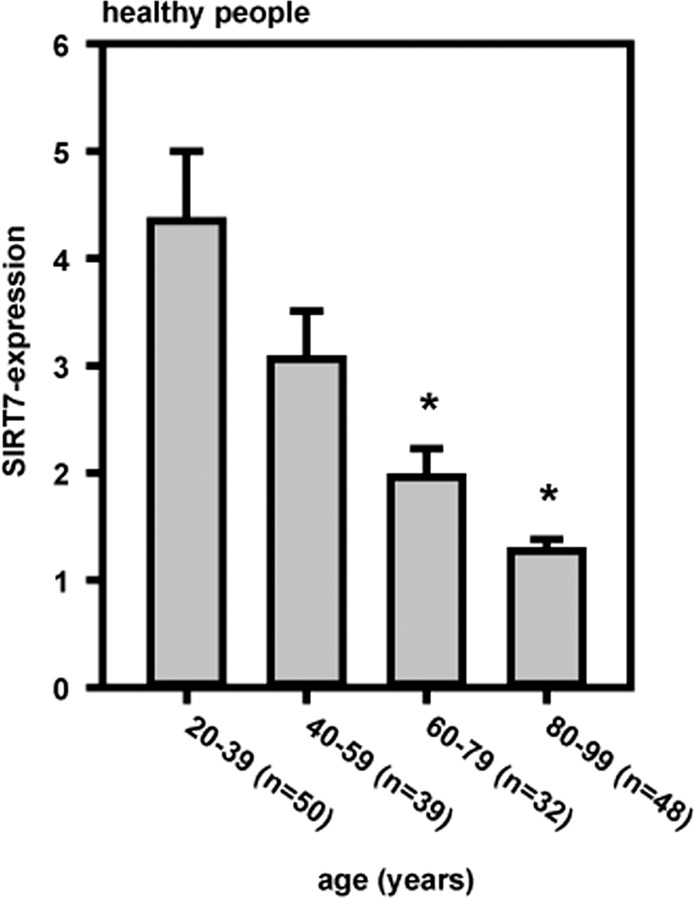
Age-dependent SIRT7-expression in blood leukocytes of healthy people. The SIRT7-expression decreased significantly depending on age (means ± SEM; significant differences corresponding to the 20–39 years age cohort (*p < 0.05); Mann–Whitney rank-sum test).

SIRT7 gene expression was measured in leukocytes of healthy people (Fig. 1). Four cohorts were distinguished, each containing a 20-year time span. SIRT7-expression decreased significantly in older cohorts. The 80–99-year-old cohort in comparison to the 20–39-year-old cohort showed an expression level of 29.3% of the initial SIRT7-expression.

### CML: SIRT7-expression and SIRT7 protein levels are influenced by BCR-ABL inhibition

**Fig. 2:**
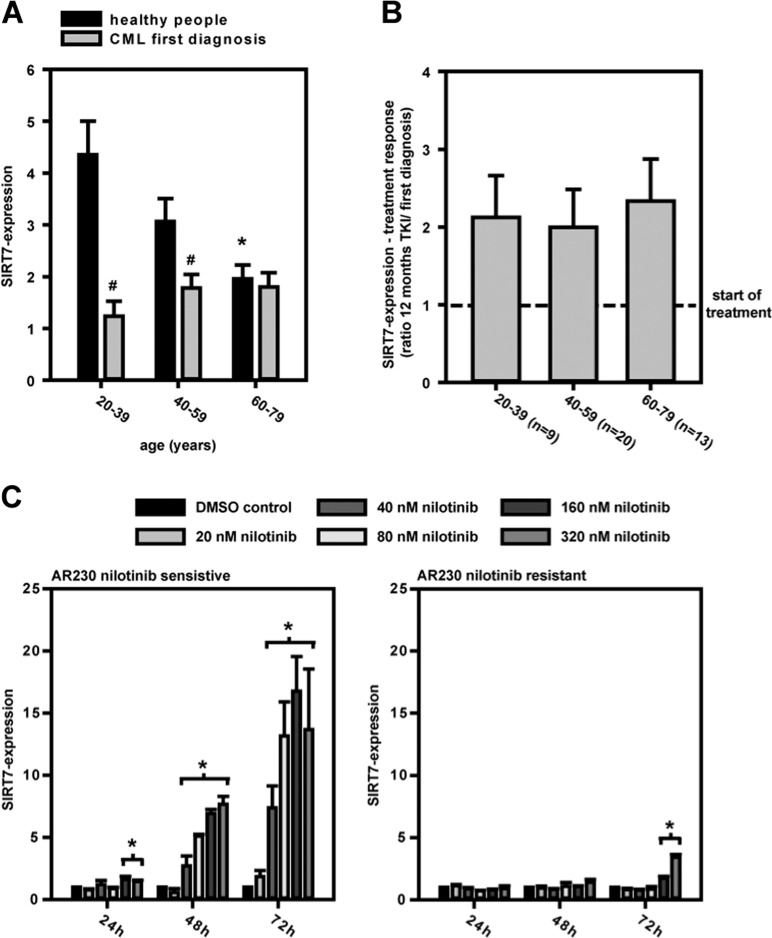
SIRT7 in CML. **a** SIRT7-expression was depressed in younger age groups with CML at diagnosis. Healthy people (*n*): 20–39 years (50), 40–59 years (39), 60–79 years (32); CML initial diagnosis: 20–39 years (17), 40–59 years (40), 60–79 years (21) (means ± SEM; Mann–Whitney rank-sum test; differences corresponding to 20–39 year age group *p < 0.05; differences between healthy people/CML initial diagnosis ^#^*p* < 0.05). **b** SIRT7-expression doubled age independently after a 12-months tyrosine kinase inhibitor (TKI; included imatinib, nilotinib, and dasatinib) treatment (means ± SEM; Mann–Whitney rank-sum test). **c** BCR-ABL in AR230 nilotinib-sensitive and -resistant cells were treated with nilotinib. SIRT7-expression was measured after 0, 24, 48, 72 h. The increased expression was associated directly with BCR-ABL inhibition, shown by the almost exclusive effect in nilotinib-sensitive AR230 cells (means ± SEM; value normalization to DMSO control; *n* = 3; Student’s *t* test (*p < 0.05)).

SIRT7-expression in leukocytes of CML patients, especially in younger patient cohorts (20–39 and 40–59 years), was significantly lower than in the leukocytes of healthy people (Fig. 2a). No differences were measured in elderly people. After BCR-ABL inhibition by tyrosine kinase inhibitors, SIRT7-expression increased in all age cohorts (Fig. 2b). SIRT7-expression also increased after BCR-ABL inhibition in CML cell lines (KCL-22, AR230) (Supplementary Fig. 3A and Fig. 2c). Clinical established tyrosine kinase inhibitors (imatinib, nilotinib, and dasatinib) showed the same effects (Supplementary Fig. 3A). Furthermore, time- and dosage-specific effects in AR230 cell lines depended directly on BCR-ABL activity as nilotinib-resistant AR230 cells showed no SIRT7-expression response to BCR-ABL inhibition by nilotinib (different mechanisms resulted in nilotinib resistance, Supplementary Table 2). In line with the RNA expression results, protein levels in western blots increased after BCR-ABL inhibition in nilotinib-sensitive AR230 cells. Principal isoform changes of SIRT7 were not seen in exon-specific PCRs or on protein levels (Supplementary Fig. 3B, C).

### AML: SIRT7-expression linked with clinical treatment response

**Fig. 3:**
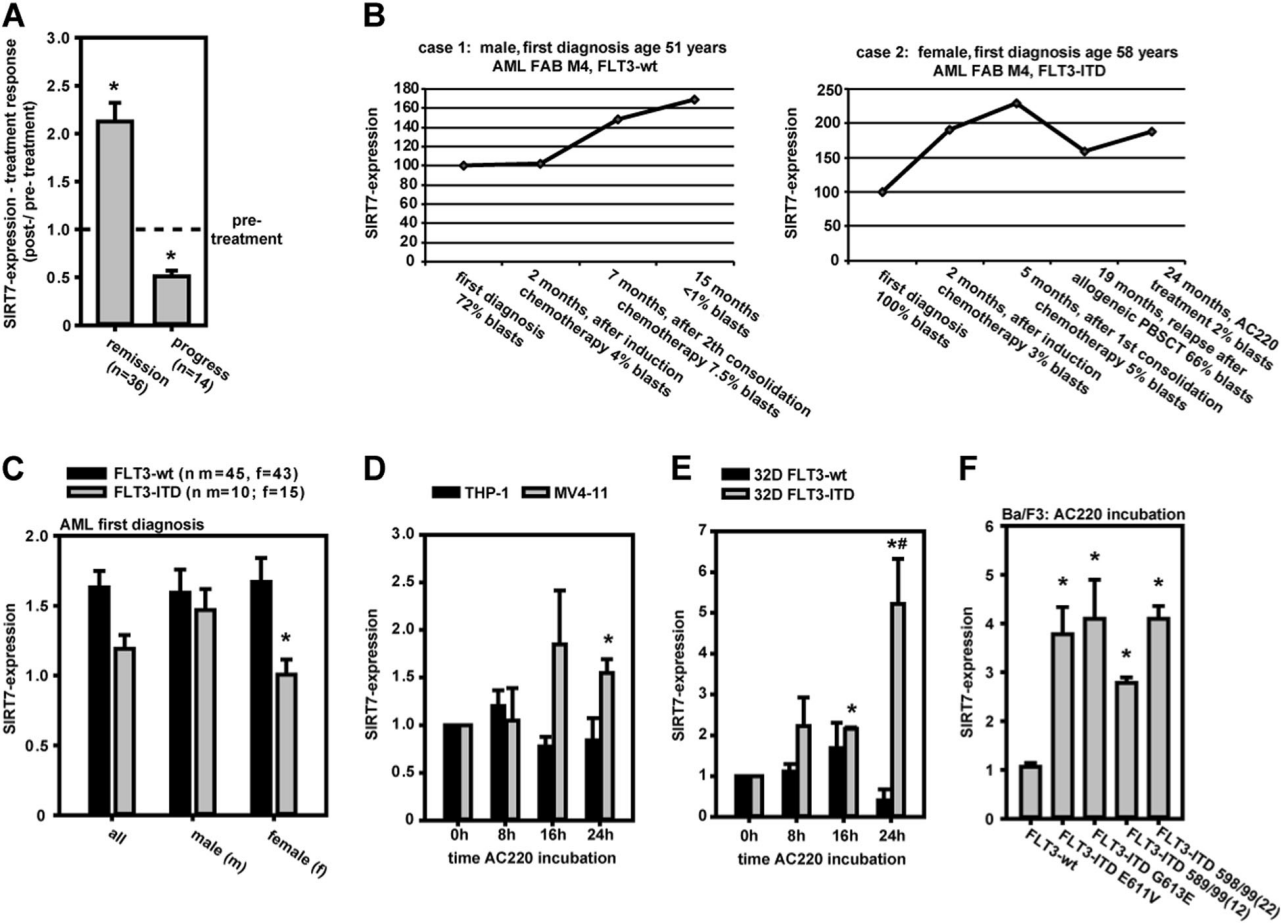
SIRT7 in AML. **a** SIRT7-expression in AML patients bone marrow depended on treatment response (mean ± SEM; Mann–Whitney rank-sum test (*p < 0.05)). **b** Clinical cases: SIRT7-expression in bone marrow corresponding to AML-treatment events in case of FLT3-wt and FLT3-ITD-positive AML is shown. **c** SIRT7-expression was significantly decreased in bone marrow leukocytes of female FLT3-ITD-positive AML patients at first diagnosis compared with wild-type patients (mean ± SEM; Mann–Whitney rank-sum test (*p < 0.05)). **d**–**f** MV4-11 (FLT3-ITD-positive) and THP-1 cells (FLT3-wt) (time course), or 32D FLT3-wt- and FLT3-ITD-cells (time course), or Ba/F3 cells (FLT3-wt or different FLT3-ITDs) (24 h) were treated by AC220. FLT3-ITD inhibition resulted in increase of SIRT7-expression (mean ± SEM; value normalization to 0 h sample (**d**, **e**) or DMSO control (**f**); *n* = 3; significant differences corresponding to 0 h (**d**, **e**), FLT3-wt (**f**) (*p < 0.05) or between FLT3-wt vs. ITD (**d**, **e**) (^#^*p* < 0.05) were identified with Student’s *t* test).

SIRT7-expression in bone marrow leukocytes of AML patients was also associated with the clinical treatment response (Fig. 3a). It increased to 212% (on average) in times of positive treatment effects (decreased percentage of myeloid blasts in bone marrow). In times of disease progress (increased percentage of myeloid blasts in bone marrow) SIRT7-expression decreased down to 51% compared with pretreatment expression. These effects were the same in FLT3-wt and FLT3-ITD mutated leukemias (Supplementary Fig. 4A). Furthermore, SIRT7-expression differed in pretreatment monitoring too (Supplementary Fig. 4B). Patients with disease progress had initial higher SIRT7-expression than patients with positive treatments effects. Two clinical case studies illustrated these effects as well (Fig. 3b). Target-based FLT3-ITD inhibition by AC220 and positive treatment response resulted in SIRT7-expression increase in case 2. Two other AC220 treated patients were identified in AML patient cohort too. In all cases positive treatment response resulted in SIRT7-expression increase (Supplementary Fig. 4C).

### AML: low SIRT7-expression associated with poor prognosis in FLT3-ITD mutated and FLT3-wild-type AML patients

Specific genetic alterations in AMLs determine favorable and unfavorable long-time patient prognosis. Especially different internal tandem duplications in FLT3 receptors (FLT3-ITD) with continuous activation of oncogenic signal transduction are associated with an unfavorable prognosis [19]. Gene expression data [20] analyzing the expression level of the mRNA encoding SIRT7 revealed that the overall survival probability of FLT3-ITD-mutated patients with a low SIRT7-expression level (*n* = 24) was significantly lower (*p* = 0.011, log-rank test) than the survival probability of FLT3-ITD-mutated patients with a high SIRT7-expression level (*n* = 57) (Supplementary Fig. 5A). Similarly, high expression of SIRT7 (*n* = 57) also correlated with the overall survival in FLT3-wild-type AML patients and revealed a significantly better survival prognosis (*p* = 0.048) compared with patients with low SIRT7-expression (*n* = 25) (Supplementary Fig. 5B). Low SIRT7-expression levels were always associated with a poorer survival prognosis. An additional FLT3-ITD mutation reduced survival probability further.

### AML: SIRT7-expression depends on FLT3-ITD mutation

SIRT7-expression measurements in bone marrow leukocytes of AML at diagnosis showed a higher SIRT7-expression in prognostic favorable FLT3-ITD-negative AML samples in comparison to FLT3-ITD-mutated AML samples. Furthermore, differences are highly significant in female subgroup. In male patients, no significant difference was measurable (Fig. 3c). The functional linkage of FLT3-ITD mechanism and SIRT7-expression regulation was also seen in cell culture experiments. FLT3-ITD inhibition by AC220 in human FLT3-ITD-mutated MV4-11 cells resulted in an increased SIRT7-expression. SIRT7-expression in FLT3-ITD-negative THP-1 cells was not affected by treatment (Fig. 3d). The similar results were found in murine 32D FLT3-WT or -ITD cells (Fig. 3e). Furthermore, inhibition of different typical FLT3-ITD mutations [21] (FLT3-ITD E611V, FLT3-ITD G613E, FLT3-ITD 589/99(12), and FLT3-ITD 598/99(22)) in murine Ba/F3 cells by AC220 supported the results generated with the other cell lines (Fig. 3f). FLT3-ITD inhibition by AC220 affected cells to increased SIRT7 mRNA-expression. Only in Ba/F3 FLT3-ITD cells AC220 induced higher normalized SIRT7 protein levels on western blots (Supplementary Fig. 4D), which was accompanied by a SIRT7-isoform shift: the 21 kDa- and 36 kDa-isoforms were suppressed, whereas the predominant 45 kDa isoform increased and a new 42 kDa-variant emerged. Such an isoform change could also be detected indirectly in the exon-specific PCR performed in human FLT3-ITD expressing MV4-11 cells (Supplementary Fig. 4E). Exon 1–5-specific PCR identified a new 450 bp product upon AC220 treatment, indicating AC220-dependent expression of an exon 1 containing (longer) SIRT7 transcript. This could be responsible for the switch from shorter to longer protein isoforms.

### SIRT7-expression increased in monocyte differentiation

**Fig. 4:**
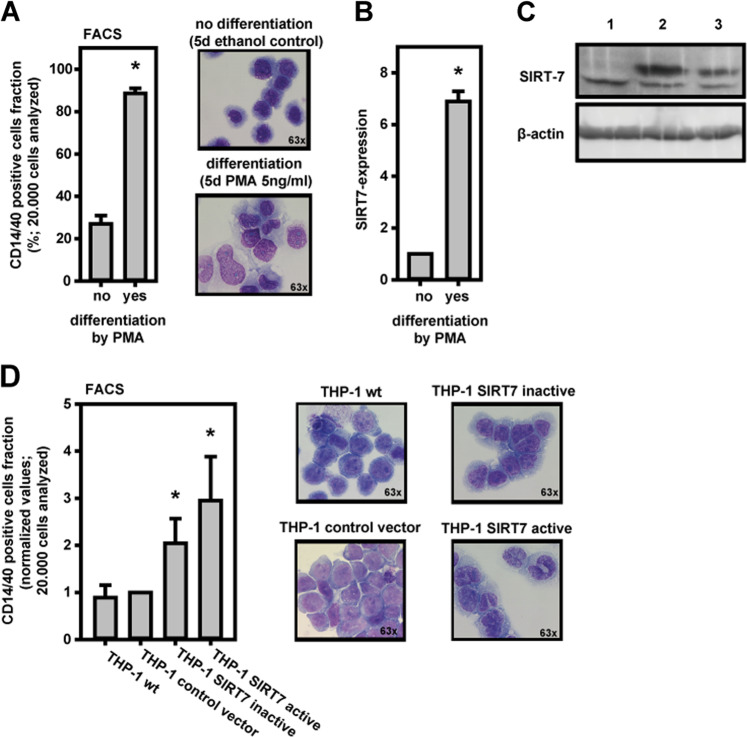
SIRT7 in THP-1 monocyte differentiation. **a** Monocyte differentiation of THP-1 cells was induced by PMA-incubation and measured in FACS by count of CD14/40^+^ cells. Monocyte cytomorphological changes were seen microscopically. **b** During PMA induced THP-1 differentiation SIRT7-expression increased. **c** THP-1 cells stably transduced with pBABE-Sirt7-WT (2; SIRT7 active), pBABE-Sirt7-HY (3; SIRT7 inactive), or empty vector (1). Whole cell lysates of THP-1 cells were subjected to SDS-PAGE, blotted to a PVDF membrane, and analyzed with antibodies recognizing SIRT7 and ß-actin as loading control. **d** The overexpression of enzymatically inactive SIRT7 and especially of active SIRT7 in THP-1 cells was associated with increased detection of CD14/40^+^ cells. Cells with active SIRT7-overexpression showed more monocytic cytomorphological features than THP-1 wt. Means ± SEM. *n* = 3. Significant differences corresponding to no differentiation (**a**, **b**) or THP-1 wt (**d**) were identified with Student’s *t* test (*p < 0.05).

THP-1 cells were differentiated to monocytes by PMA-incubation. Successful differentiation effects were measured by FACS analysis (increase of CD14/40-positive cells) (Fig. 4a). Furthermore, typical monocyte differentiation effects were monitored by cytomorphological changes in microscopy. In time of differentiation SIRT7-expression increased (Fig. 4b).

Furthermore, overexpression of SIRT7 in THP-1 cells was also associated with differentiation. THP-1 cells were stably transduced with expression constructs encoding wild-type SIRT7 or enzymatically inactive SIRT7 H187Y [15] (Fig. 4c). Overexpression of SIRT7 resulted in stimulation of cell differentiation as demonstrated by increase of CD14/40-positive cells and pronounced monocyte-typical morphological changes (Fig. 4d). Interestingly, overexpression of inactive SIRT7 H187Y also resulted in partial stimulation of THP-1 differentiation indicating a noncatalytic function of SIRT7 (Fig. 4d).

### C/EBPα, -β, and -ε: missing links in FLT3-ITD/SIRT7, BCR-ABL/SIRT7, and monocyte differentiation/SIRT7 relationship

**Fig. 5:**
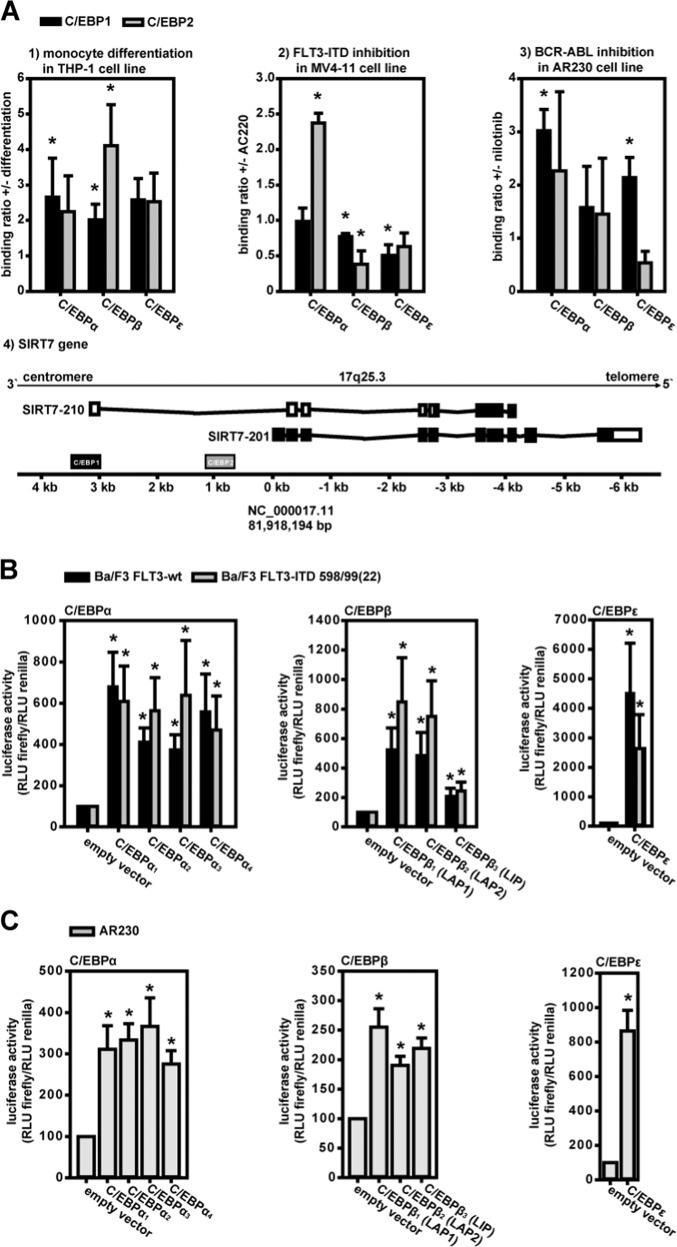
C/EBPα, -β, and -ε: missing links in FLT3-ITD/SIRT7, BCR-ABL/SIRT7, and monocyte differentiation/SIRT7 relationships. **a** Inhibition- or differentiation-dependent binding of C/EBPα, -β, and -ε to putative C/EBP-binding sites (C/EBP1 and C/EBP2) within the human SIRT7 promoter (**a4**) was analyzed by ChIP. Putative C/EBP-binding sites are located near the TSS of human SIRT7-210 and SIRT7-201 isoforms (protein-coding isoforms, derived from Ensembl Genome Browser). **a1** Monocyte differentiation of THP-1 cells was induced by PMA. **a2** FLT3-ITD in MV4-11 cells was inhibited with AC220. **a3** BCR-ABL in AR230 cells was inhibited by nilotinib (mean ± SEM; *n* = 3; Student’s *t* test (*p < 0.05); differences corresponding to no differentiation (**a1**) or inhibitor treatment (**a2**, **a3**); no difference mean ratio 1). **b**, **c** C/EBP-isoform dependent SIRT7-promoter activity was analyzed in reporter-gene assays. C/EBPα or -β isoforms and full-length C/EBPε were overexpressed in Ba/F3 cells with FLT3-wt or FLT3-ITD 598/99(22) and in AR230 cells, resulting in increased promoter activities. The activating effect of C/EBPβ_3_ (LIP) was lower than that of LAP isoforms in Ba/F3 cells (means ± SEM; *n* = 3; Student’s *t* test (*p < 0.05); differences corresponding to empty vector controls).

C/EBPα, -β, and -ε proteins regulate many transcriptional mechanisms in hematopoiesis and stem-cell differentiation. C/EBP dysregulation is often associated with hematopoietic stem-cell disorders showing disturbed cell differentiation [22–27]. Therefore, we investigated the influence of C/EBPs on SIRT7-expression. MatInspector database search results revealed 2 putative C/EBP-binding sites in the SIRT7 gene promoter region (C/EBP1: NC_000017.11 (81,921,507–81,921,521); C/EBP2: NC_000017.11 (81,919,160–81,919,174)). The ChIP results confirmed C/EBPα, -β, and -ε binding to both C/EBP-binding sites in the SIRT7-promoter region (Fig. 5a). In the THP-1 monocyte differentiation model all C/EBPs revealed increased binding to both binding sites after induction of differentiation. In MV4-11 FLT3-ITD-AML cells C/EBPα binding to C/EBP2 increased after FLT3-ITD inhibition. C/EBPα binding to C/EBP1 and C/EBPβ/ε binding to C/EBP1/2 were unaffected or reduced. In AR230 CML cells C/EBP binding increased after BCR-ABL inhibition by nilotinib (with the exception of C/EBPε-binding to C/EBP2).

BCR-ABL inhibition in AR230 cells (Supplementary Fig. 6A) or induction of cell differentiation in THP-1 cells (Supplementary Fig. 6B) resulted in higher C/EBPα, -β, and -ε gene expression (whereas AC220 treatment of THP-1 cells did not; Supplementary Fig. 6C). FLT3-ITD inhibition in MV4-11 and Ba/F3 FLT3-ITD-positive cells showed somewhat different results (Supplementary Fig. 6C, D). Here, C/EBPα and -ε expression increased, but C/EBPβ decreased or was unaffected. However, protein isoform analyses in western blots of murine Ba/F3 cells relativized mRNA-expression findings (Supplementary Fig. 6E). Only the inhibitory C/EBPβ-LIP isoform protein level was reduced in FLT3-ITD expressing Ba/F3 cells. The activating C/EBPβ-LAP1 isoform was not reduced. All in all, there is a relative increase in the activating C/EBPβ isoform (previously described by Haas et al. [25]).

Finally, all C/EBPα and -β isoforms and full-length C/EBPε were overexpressed in Ba/F3 FLT3-wt, Ba/F3 FLT3-ITD 598/599(22)-AML cells, and AR230 CML cells, which were co-transfected with a C/EBP1/2 binding-site containing SIRT7-promoter-luciferase reporter-gene plasmid. In the AML-cell model (Fig. 5b), all C/EBPs induced luciferase activity in FLT3-ITD-mutated and wild-type Ba/F3 cells. The strongest effect was measured in case of C/EBPε-overexpression. The weakest inducer was C/EBPβ-LIP. In the CML cell model (Fig. 5c), all C/EBPs significantly upregulated luciferase activity in BCR-ABL AR230 cells. The strongest effect again was induced by C/EBPε-overexpression. In general, C/EBP-overexpression reversed the effects of the mutations.

## Discussion

This study identified an age-dependent decrease of SIRT7 gene expression in healthy leukocytes. SIRT7-expression in humans was age dependent, as observed in the mouse model. Mohrin et al. showed that low SIRT7 gene expression levels were typical in older adult murine HSCs. SIRT7 downregulation was associated with myeloid cell proliferation and reduced longevity [5–7].

Older humans have a higher risk of developing myeloid stem-cell disorders, such as the well-known age-related leukemias AML and CML. It was therefore hypothesized that SIRT7 gene expression might be reduced at the time of disease diagnosis. Conversely, expression should increase as a result of positive treatment response when hematopoiesis reaches normal conditions and pathomechanisms, which lower SIRT7-expression are inhibited. SIRT7 could be a tumor suppressor and a biomarker to evaluate the treatment effects in myeloid stem-cell disorders. To confirm SIRT7 functions as a tumor suppressor additional experiments are needed.

The results presented here confirm this theory. SIRT7-expression levels at the time of CML and AML diagnosis were significantly lower than in healthy young people. Furthermore, expression levels improved after successful targeted treatment. In cases of relapse or disease progress SIRT7-expression levels in AML decreased significantly. Therefore, after further clinical evaluation SIRT7 may serve as a useful biomarker to control the treatment outcome in myeloid stem-cell disorders in correlating with positive and negative treatment responses.

In CML- and FLT3-ITD-mutated AML-cell lines SIRT7 gene expression increase was seen after successful specific treatment. Direct comparison of BCR-ABL inhibition in nilotinib-sensitive and -resistant AR230 CML cells verified direct association to BCR-ABL-specific effects. SIRT7-expression only increased in nilotinib-sensitive cells after BCR-ABL inhibition. Therefore, the detectable effects on SIRT7-expression in patient samples are likely to depend directly on BCR-ABL activity.

Driver mutation dependency was also found in AML. The activity of typical FLT3-ITD mutations affected SIRT7-expression levels in the Ba/F3 cell line model. After inhibition of FLT3-ITD activity by AC220 SIRT7-expression increased.

Interestingly, on the protein level SIRT7-isoform changes were associated with AC220 treatment effects. FLT3-ITD inhibition in MV4-11 cells induced a new unknown 42 kDa isoform of SIRT7. This could be explained by a change in exon usage by, e.g., exon skipping or altered transcription initiation points. Some indications for such mechanisms were detected through exon-specific PCRs of the SIRT7 gene. A new 450 bp PCR-fragment was found in exon 1–5-specific PCR. This points toward versatile transcriptional and maybe translational regulatory processes involved in isoform shifting. The molecular reason is currently not understood.

FLT3-ITD-dependent reduction of SIRT7-expression was also found in bone marrow samples of AML patients at diagnosis. Interestingly, the effect was more dominant in female patients. Influence of female or male hormones on leukemia development and treatment has already been described [28–31]. But detailed mechanisms are unknown. The AC220 effect on SIRT7 was also evident in a case report: AC220 treatment of an AML relapse with FLT3-ITD mutation after allogeneic stem-cell transplantation resulted in increased SIRT7-expression. Therefore, FLT3-ITD-activity is one important regulator of SIRT7 gene expression.

Next to the direct influence of driver mutations on SIRT7-expression, subgroup analyzes of established gene expression data [20] correlated reduced SIRT7 gene expression with poor prognosis in FLT3-ITD-mutated and FLT3-wild-type AML patients. FLT3-ITD mechanisms are one important part in SIRT7-expression regulation. But data revealed an influence in FLT3-wt leukemias too. Therefore, there have to be other unknown mechanisms next to the important FLT3-ITD pathway.

Our results suggest that SIRT7 gene expression is likely regulated by a disease entity-overlapping mechanism. Low SIRT7 levels were always associated with active disease (nonhealthy conditions), in both BCR-ABL- and FLT3-ITD-positive leukemias. In case of a positive treatment response, SIRT7-expression levels increased (healthy condition). In clinical practice a normalized hematopoiesis resulted in better cell maturation and differentiation. The relationship of high SIRT7-expression levels and cell differentiation, especially monocyte differentiation, was investigated.

Monocyte differentiation of THP-1 cells by PMA resulted in increased SIRT7 gene expression. Induced SIRT7-overexpression also affected cell differentiation, as cytomorphological evaluation showed development toward the monocytic phenotype and induction of CD14/40-positive cells. The strong association with cell differentiation points toward SIRT7 having a role as a monocyte cell differentiation factor. Interestingly, overexpression of catalytically inactive SIRT7 H187Y also partially stimulated cell differentiation pointing a noncatalytic function of SIRT7 in this process. A function of enzymatically inactive SIRT7 has been demonstrated earlier [32]. This observation serves as a basis for our further investigations.

One general well-known factor of regulation of gene expression in myeloid stem-cell disorders is the influence of the transcription factor C/EBPα [22]. Functions of C/EBPα can be inhibited by BCR-ABL-induced upregulation of hRNP-E2 with associated C/EBPα mRNA instability in CML and inhibition of C/EBPα dimerization by FLT3-ITD-induced phosphorylation in AML. Furthermore, C/EBPα influences transcriptional control of myeloid cell differentiation [23].

The C/EBP transcription factor family is encoded by six genes, namely C/EBPα, C/EBPβ, C/EBPε, C/EBPγ, C/EBPδ, and C/EBPζ [33]. The most relevant C/EBPs in myeloid cell differentiation are C/EBPα, C/EBPβ, and C/EBPε [23]. C/EBP homo- or heterodimers bind to typical C/EBP *cis*-elements in promoter regions and influence transcriptional activity [33]. Therefore, the roles of C/EBPα, C/EBPβ, and C/EBPε in SIRT7-promoter regulation were investigated. MatInspector database searches revealed two putative C/EBP-binding sites in the SIRT7-promoter region. ChIP experiments proved binding of all investigated C/EBP variants to these sites. In THP-1 cells, monocyte-typic differentiation increased C/EBP binding. Similar results were found in AR230 CML cells after BCR-ABL inhibition. Only C/EBPε binding decreased. In MV4-11 AML cells FLT3-ITD inhibition by AC220 resulted in increased C/EBPα binding. C/EBPβ and C/EBPε binding decreased after inhibition. C/EBP-specific effects were supported by the results of reporter-gene assays when C/EBP-isoforms were overexpressed. All C/EBPs activated the SIRT7 promoter. The strongest inductor was C/EBPε. The weakest inductor was the well-known inhibitory isoform of C/EBPβ called C/EBPβ-LIP. Furthermore, cell-specific increases of C/EBP-expression corresponded with the ChIP results.

These results indicate a positive involvement of C/EBPs in regulation of SIRT7-expression. However, cell-specific differences were observed in ChIP experiments. Overall, C/EBPα activated SIRT7-expression in all cell models.

In contrast to our results Liu et al. characterized C/EBPα as an inhibitor of SIRT7-expression in hepatocellular carcinoma cells [34]. Furthermore, SIRT7 is upregulated in the majority of cancers [35], including colorectal [36], gastric [37], breast [38, 39], bladder [40], ovarian [41], cervical [42], and hepatocellular [43] cancers. High SIRT7-expression is a predictor of poor survival in various cancers. SIRT7 acts in these cases like an oncogene and not like a tumor suppressor. But conversely, Mc Glynn et al. reported a tumor-suppressive effect of SIRT7 in pancreatic cancers [44]. Furthermore, Hubbi et al. described tumor suppressor effects of SIRT7 by negative regulation of HIF1 and HIF2 transcription [32]. All examples show that SIRT7 mediates ambivalent effects in tumor biology. It can act as an oncogene or tumor suppressor in cell-specific ways. Therefore, C/EBPα could inhibit SIRT7-expression in hepatocellular carcinoma cells [34], yet activate SIRT7-expression in hematopoietic cells.

In summary, the intracellular SIRT7-level in human hematopoietic cells was age dependent. The results were consistent with Mohrin’s data [5]. Low SIRT7 levels were associated to age-dependent malignant myeloid stem-cell disorders. Furthermore, successful treatment of CML or AML increased SIRT7 levels. SIRT7 levels increased during hematopoietic cell differentiation. SIRT7-expression was directly influenced by effects of specific driver mutations (BCR-ABL in AML, FLT3-ITD in AML). The activating effects of C/EBP transcription factors (described in detail by Tsukada [45] and Avellino [22]) might explain the relationship between driver mutations effects, cell differentiation, and SIRT7-expression.

Taken together, SIRT7 is an important element in human hematopoietic cell aging and longevity. Furthermore, it is an important tumor suppressor and could serve potentially as a biomarker for monitoring the clinical treatment response in myeloid stem-cell disorders AML and CML.

### Supplementary information

Supplemental Material
