# Publisher Correction: SIRT7: an influence factor in healthy aging and the development of age-dependent myeloid stem-cell disorders

**DOI:** 10.1038/s41375-021-01365-4

**Published:** 2021-11-16

**Authors:** Alexander Kaiser, Martin Schmidt, Otmar Huber, Jochen J. Frietsch, Sebastian Scholl, Florian H. Heidel, Andreas Hochhaus, Jörg P. Müller, Thomas Ernst

**Affiliations:** 1 Klinik für Innere Medizin II, Abteilung Hämatologie und Internistische Onkologie, Universitätsklinikum Jena, Jena, Germany; 2 Institut für Biochemie II, Universitätsklinikum Jena, Friedrich-Schiller-Universität, Jena, Germany; 3 Leibniz-Institute on Aging (Fritz-Lipmann-Institute), Jena, Germany; 4 Institut für Molekulare Zellbiologie, CMB, Universitätsklinikum Jena, Friedrich-Schiller-Universität, Jena, Germany

**Keywords:** Leukaemia, Cancer stem cells

Correction to: *Leukemia* 10.1038/s41375-020-0803-3

The article SIRT7: an influence factor in healthy aging and the development of age-dependent myeloid stem-cell disorders, written by Alexander Kaiser, Martin Schmidt, Otmar Huber, Jochen J. Frietsch, Sebastian Scholl, Florian H. Heidel, Andreas Hochhaus, Jörg P. Müller and Thomas Ernst, was originally published Online First without Open Access. After publication in volume 34, page 2206-2216, the author decided to opt for Open Choice and to make the article an Open Access publication. Therefore, the copyright of the article has been changed to © The Author(s) 2020 and the article is forthwith distributed under the terms of the Creative Commons Attribution.

