# Supplementary material for: SIRT7: an influence factor in healthy aging and the development of age-dependent myeloid stem-cell disorders
Source: Leukemia. 2020 Mar 25;34(8):2206–16. doi: 10.1038/s41375-020-0803-3 (PMC8318878; doi:10.1038/s41375-020-0803-3)
Supplement: Supplementary file 1 — Supplemental Material [file 41375_2020_803_MOESM1_ESM.pdf]

Supplemental Data

SIRT7: An influence factor in healthy aging and the development of age dependent myeloid stem cell disorders

Alexander Kaiser[1], Martin Schmidt[2], Otmar Huber[2], Jochen J Frietsch[1], Sebastian Scholl[1], Florian H Heidel[1,3], Andreas Hochhaus[1], Jörg P Müller[4*], Thomas Ernst[1*]

[1]Klinik für Innere Medizin II, Abteilung Hämatologie und Internistische Onkologie, Universitätsklinikum Jena, Germany

[2]Institut für Biochemie II, Universitätsklinikum Jena, Germany, Friedrich-Schiller-Universität, Jena, Germany

[3]Leibniz-Institute on Aging (Fritz-Lipmann-Institute), Jena, Germany

[4]Institut für Molekulare Zellbiologie, CMB, Universitätsklinikum Jena, Friedrich-Schiller-Universität, Jena, Germany

*shared senior authorship

Supplement

Supplement - Methods

	name	Primer sequences	Denaturation	Annealing	Elongation	Cycles
**qRT-PCR** (initial denaturation 2 min, 95°C)	hβ-glucuronidase(β-GUS)[1]	F: 5´- AGAAACGATTGCAGGGTTTCAC-3´ R: 5´- CCGAGTGAAGATCCCCTTTTTA -3´	95°C 15s	55,5°C 30 s	72°C 25 s	43
	mβ-actin[2]	F: 5´- AGCCATGTACGTAGCCATCC-3´ R: 5´- CTCTCAGCTGTGGTGGTGAA-3´	95°C 15s	55,5°C 30 s	72°C 25 s	43
	hSIRT7[3]	F: 5´-CGCCAAATACTTGGTCGTCT-3´ R: 5´-CCCTTTCTGAAGCAGTGTCC-3´	95°C 15s	55,5°C 30 s	72°C 25 s	43
	mSIRT7[3]	F: 5´-CCATGGGAAGTGTGATGATG-3´ R 5´-TCCTACTGTGGCTGCCTTCT -3´	95°C 15s	55,5°C 30 s	72°C 25 s	43
	hC/EBPα[4]	F: 5´- CGGTGGACAAGAACAGCAAC-3´ R: 5´- CGGAATCTCCTAGTCCTGGC-3´	95°C 15s	55,5°C 30 s	72°C 25 s	43
	mC/EBPα[5]	F: 5´- CGGTGCGCAAGAGCCGAGAT-3´ R: 5´- CCCGCAGCGTGTCCAGTTCA-3´	95°C 15s	55,5°C 30 s	72°C 25 s	43
	hC/EBPβ	F: 5´- CGCAGGTCAAGAGCAAGG-3´ R: 5´- GCTGCTTGAACAAGTTCCGC-3´	95°C 15s	55,5°C 30 s	72°C 25 s	43
	mC/EBPβ[4]	F: 5´- GTTTCGGGACTTGATGCAAT-3´ R: 5´- CCCCGCAGGAACATCTTTA-3´	95°C 15s	55,5°C 30 s	72°C 25 s	43
	hC/EBPε[6]	F: 5´-CAGCCGAGGCAGCTACAATC-3´ R: 5´- AGCCGGTACTCAAGGCTATCT-3´	95°C 15s	55,5°C 30 s	72°C 25 s	43
	mC/EBPε[7]	F: 5´-CTGGGGAAGAACAGCTACTTTC-3´ R: 5´-GTGAGGGATAGGCGAATGGC-3´	95°C 15s	55,5°C 30 s	72°C 25 s	43
**ChIP**	C/EBP1	F: 5´- TACGGACACCTCTTTGTGGC-3´ R: 5´- GCTGCACAAAACCTCACCAG -3´	95°C 15s	60°C 30 s	72°C 30 s	40
	C/EBP2	F: 5´- CCGCTCTGGGATCTTGTGAG-3´ R: 5´- CTCTACTCTCCCGACCTGCT -3´	95°C 15s	60°C 30 s	72°C 30 s	40
**Exon-specific PCR** (combinations Ex5-rev with Ex1-, Ex2-, Ex3-, Ex3alt-for or Ex4-for with Ex6-, Ex7-, Ex8. Ex9, Ex10-rev) (initial denaturation 2 min, 95°C)	hSIRT7-Ex5-rev	R: 5´- GGTGATGCTCATGTGGGTGA-3´	95°C 15s	60°C 30 s	72°C 30 s	40
	hSIRT7-Ex1-for	F: 5´- GGAGGAGCAGCAGAGGGA -3´	95°C 15s	60°C 30 s	72°C 30 s	40
	hSIRT7-Ex2-for	F: 5´- GAGAGCGCGGACCTGGTAA-3´	95°C 15s	60°C 30 s	72°C 30 s	40
	hSIRT7-Ex3-for	F: 5´- ACTTGGTCGTCTACACAGGC-3´	95°C 15s	60°C 30 s	72°C 30 s	40
	hSIRT7-Ex3alt-for	F: 5´- TACCCCAGGACGGAGTATGA-3´	95°C 15s	60°C 30 s	72°C 30 s	40
	hSIRT7-Ex4-for	F: 5´- AGGGAGAAGCGTTAGTGCTG-3´	95°C 15s	60°C 30 s	72°C 30 s	40
	hSIRT7-Ex6-rev	R: 5´- CAGTTCTGAGACACCACATGCT-3´	95°C 15s	60°C 30 s	72°C 30 s	40
	hSIRT7-Ex7-rev	R: 5´- GGAACGCAGGAGGTACAGAC-3´	95°C 15s	60°C 30 s	72°C 30 s	40
	hSIRT7-Ex8-rev	R: 5´- CAGAGGCGTGGGTACTTCTTT-3´	95°C 15s	60°C 30 s	72°C 30 s	40
	hSIRT7-Ex9-rev	R: 5´- AGTCATCCTTCGGGGTCCA-3´	95°C 15s	60°C 30 s	72°C 30 s	40
	hSIRT7-Ex10-rev	R: 5´- GGGAGTCGCCAGTGAGAAAA-3´	95°C 15s	60°C 30 s	72°C 30 s	40

Supplementary table 1. Oligonucleotides (primers) and PCR conditions.

h, human; m, murine; Ex, exon. qRT-PCR: The reaction set up (10 µl) was as follows: 1 µl cDNA, 0.5 µM of each primer and 1 × FastStart SYBR Green Master (Roche, Mannheim, Germany). All reactions were set up in duplicate per experiment. Evaluation of qRT-PCR results was done by the $\Delta\Delta C_T$-method[8].

Exon-specific PCR: Analysis of expressed SIRT7 exons

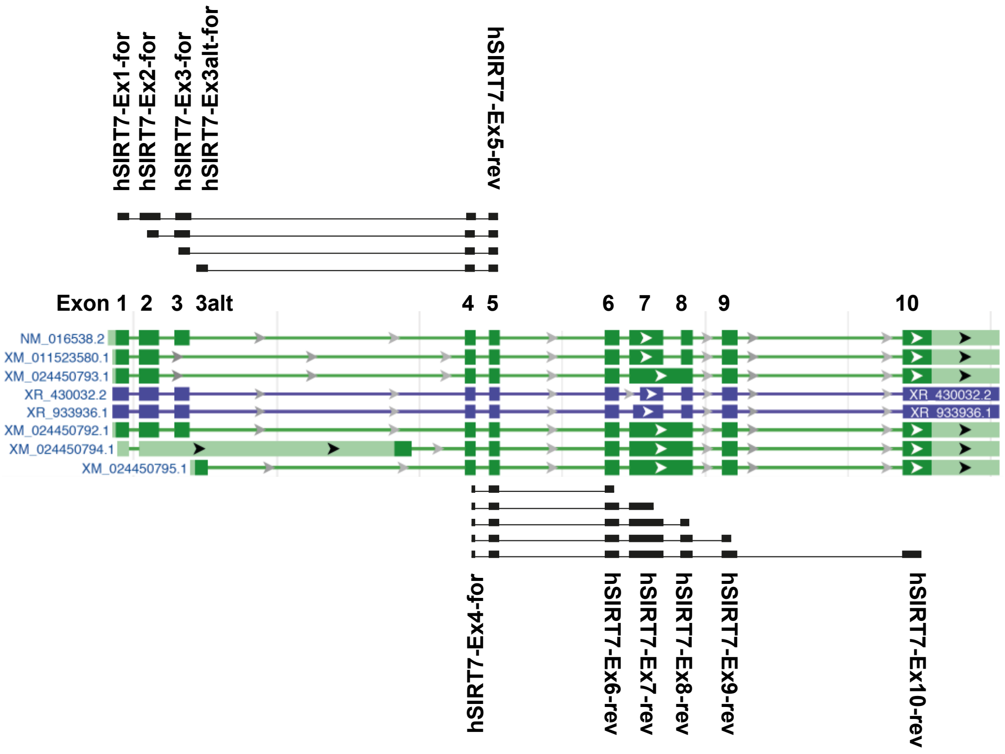

Supplementary figure 1. Analysis of expressed SIRT7-exons.

Transcription of all putative exons of the human SIRT7-gene was analyzed by reverse transcription-PCR (RT-PCR). RNA was isolated using the RNeasy Mini Kit (Qiagen, Hilden, Germany). Subsequently, cDNA was synthesized with the High-Capacity cDNA Archive Kit (Applied Biosystems) using random hexameric primers. PCR-amplifications were done in a StepOnePlus Real-Time PCR system (Applied Biosystems) using the Absolute SybrGreen Rox Mix (Promega). Initial denaturation for 2 min at 95 °C was followed by 40 cycles of 15 s at 95 °C, 30 s at 60 °C and 30 s at 72 °C. Primers were designed as to generate overlapping sets of amplicons covering all 10 exons of SIRT7 (GenBank-entry NM_016538.3). In addition a primer (hSIRT7-Ex3alt-for) was used, which allows detection of the alternative first exon of GenBank-entry XM_024450795.1, thus ensuring that all exons of putative transcripts associated with the GRCh38.p12 primary assembly of the human reference genome were covered. Primer sequences are given in Supplementary table 1. PCR-products were evaluated in 2% (w/v) agarose gels. All conditions were tested in duplicate per experiment in two independent experiments. The graphics showing SIRT7 gene organization was taken

from the NCBI GenBank gene browser (NC_000017.11 Reference GRCh38.p12 Primary Assembly). The two sets of expected transcripts resulting from the different primer combinations are indicated in black. Forward (-for) and reverse primers (-rev) are indicated by primer names.

Disease	Cell line	Origin	Cell culture conditions
CML	KCL-22	German Collection of Microorganisms and Cell Cultures (DSMZ, Braunschweig, Germany)	RPMI 1640 with 2% l-glutamine (RPMI, GIBCO-BRL, Karlsruhe, Germany) and 10% heat-inactivated fetal-bovine serum (FCS, GIBCO-BRL)
	Nilotinib-sensitive AR230	provided by François Xavier Mahon[9]	RPMI 1640 with 2% l-glutamine (RPMI, GIBCO-BRL, Karlsruhe, Germany) and 10% heat-inactivated fetal-bovine serum (FCS, GIBCO-BRL)
	Nilotinib-resistant AR230 (resistance mediated by different mechanisms[9])		
AML	THP-1 (FLT3-wt)	German Collection of Microorganisms and Cell Cultures (DSMZ, Braunschweig, Germany)	RPMI 1640 with 2% l-glutamine (RPMI, GIBCO-BRL, Karlsruhe, Germany) and 10% heat-inactivated fetal-bovine serum (FCS, GIBCO-BRL)
	MV4-11 (FLT3-ITD)	German Collection of Microorganisms and Cell Cultures (DSMZ, Braunschweig, Germany)	RPMI 1640 with 2% l-glutamine (RPMI, GIBCO-BRL, Karlsruhe, Germany) and 10% heat-inactivated fetal-bovine serum (FCS, GIBCO-BRL)
	32D cell line with murine FLT3-wt	provided by Rebekka Grundler[10]	RPMI 1640 with 2% l-glutamine (RPMI, GIBCO-BRL, Karlsruhe, Germany) and 10% heat-inactivated fetal-bovine serum (FCS, GIBCO-BRL) + murine interleukin 3 (10 ng/ml; Immunotools, Friesoythe, Germany)
	32D cell line with murine FLT3-ITD		
	Ba/F3 cell line with human FLT3-wt	provided by Florian Heidel[11]	RPMI 1640 with 2% l-glutamine (RPMI, GIBCO-BRL, Karlsruhe, Germany) and 10% heat-inactivated fetal-bovine serum (FCS, GIBCO-BRL) + murine interleukin 3 (10 ng/ml; Immunotools, Friesoythe, Germany)
	Ba/F3 cell line with human FLT3-ITD		
	Ba/F3 cell line with human FLT3-ITD G613E		
	Ba/F3 cell line with human FLT3-ITD 589/99(12)		
	Ba/F3 cell line with human FLT3-ITD 598/99(22)		
otherwise	HEK293T cells	German Collection of Microorganisms and Cell Cultures (DSMZ, Braunschweig, Germany)	DMEM (GIBCO-BRL) supplemented with 10% fetal heat-inactivated FCS

Supplementary table 2. Cell lines and cell culture conditions.

All cells were cultured in a 5% CO_2 air environment and 37°C fully humidified incubator. DMEM, Dulbecco modified Eagle medium; FLT3-wt, FLT3-wild type; RPMI, Roswell Park Memorial Institute medium.

	Antigen	Antibody name	Dilution	Origin
Western blotting	SIRT7	rabbit anti-SIRT7 (ab62748)	1:1000	abcam, Cambridge, UK
	C/EBPβ	mouse anti-C/EBPβ (H-7) (sc-7962)	1:1000	Santa Cruz Biotechnology, Dallas, USA
	β-actin	mouse anti-β actin (AC15) (A5441)	1:5000	Sigma-Aldrich, Deisenhofen, Germany
	GAPDH	mouse anti-GAPDH (6C5) (MAB374)	1:5000	Merck Millipore, Burlington, USA
FACS	CD14	APC mouse anti-human CD14 (clone MφP9)	1:5	BD Bioscience, Heidelberg, Germany
	CD40	PE mouse anti-human CD40 (clone 5C3)	1:5	BD Bioscience, Heidelberg, Germany
ChIP	C/EBPα	mouse anti-C/EBPα (G-10) (sc-166258)	-	Santa Cruz Biotechnology, Dallas, USA
	C/EBPβ	mouse anti-C/EBPβ (H-7) (sc-7962)	-	Santa Cruz Biotechnology, Dallas, USA
	C/EBPε	mouse anti-C/EBPε (C-10) (sc-515192)	-	Santa Cruz Biotechnology, Dallas, USA

Supplementary table 3. Antibodies.

SIRT7 overexpression: Production of pseudoviral particles and cell transduction

For the production of retroviral particles, HEK-293T cells were transfected with plasmids encoding FLAG-tagged SIRT7 WT (pBABE-SIRT7 WT) or enzymatically inactive SIRT7 (pBABE-SIRT7 $H_{187}Y$)[12, 13]. Co-transfection was carried out with pMDLg/pRRE and pCMV-VSV-G for production of retroviral pseudoparticles. Transfection was done using the polyethylenimine transfection reagent. The viral particles were harvested at 24, 48 and 72 hours after transfection by filtering the medium through syringe filter units (0.45 µm). Viral particles were concentrated 20-fold using an Amicon Ultra-15 filter unit (cut-off 30 kDa). Subsequently, THP-1 cells were transduced three times with the pseudotyped particles in the presence of 8 µg/ml polybrene (1,5-dimethyl-1,5-diazaundecamethylene polymethobromide, Sigma-Aldrich). Selection of cells with 2 µg/ml puromycin was started 48 hours after transduction.

Plasmids

Plasmids pFLAG-CMV4-hCEBPα_1, -α_2, -α_3 and -α_4 for expression of the four known variants of human C/EBPα were generated from a clone harboring a synthetic gene encoding the longest human protein variant (Supplementary figure 2). EcoRI-XhoI-fragments from respective pGEX4T1-hC/EBPβ expression-vectors[14] were subcloned into pcDNA3.1-HisC to generate pcDNA3.1-HisC-hCEBPβ_1, -β_2 and -β_3. These plasmids were provided by Jose L. Gutierrez (Concepcion, Chile). Plasmid pCMV6-Entry-mCEBPε for expression of full-length murine C/EBPε was obtained from OriGene (MR217079; Rockville, MD, USA). A reporter-gene plasmid, pGL3P-SIRT7-promoter, containing a 3400 bp-fragment containing the SIRT7 promoter-region as identified with the Ensembl Genome Browser was obtained by subcloning a KpnI-NheI-fragment from pGH-Amp-SIRT7-Promotor into the pGL3-promoter vector. pGH-Amp-SIRT7-promotor contained the region from positions 81921600 to 81918201 (5´-3´) of the NC_000017.11 Reference GRCh38.p12 Primary Assembly and was made by gene synthesis (Eurofins Genomics, Ebersberg, Germany). All plasmids were verified by sequencing.

A

GAATTCAATGAGAGGGAGGGGACGAGCAGGGAGTCCTGGGGGAAGAAGGAGACGTCCTGCTCAGGCTGGA

EcoRI *TSS1*

GGTCGAAGAGGTTCCCCCTGCCGTGAGAACTCCAATTCCCCGATGGAGTCAGCCGACTTTTACGAAGCCG

TSS2

AGCCTAGGCCTCCGATGAGCTCACACCTGCAATCTCCTCCCCACGCACCCTCGAGCGCTGCATTTGGCTT

AvrII *TSS3* *XhoI*

TCCACGGGGTGCAGGCCCTGCTCAACCTCCAGCTCCACCTGCTGCACCTGAACCCCTTGGGGGCATTTGT

GAGCACGAGACGAGCATCGACATCAGCGCCTACATAGACCCCGCCGCGTTCAACGACGAGTTCCTGGCCG

ATCTGTTCCAGCACAGCAGACAGCAGGAGAAGGCTAAGGCTGCAGTGGGACCTACAGGTGGCGGAGGTGG

CGGTGATTTTGATTACCCGGGGGCACCTGCAGGACCTGGAGGCGCTGTTATGCCAGGGGGTGCGCACGGA

TSS4 *FspAI*

CCTCCACCTGGTTACGGGTGTGCAGCTGCAGGCTATCTCGACGGACGCCTCGAACCCCTGTACGAAAGGG

TTGGGGCACCGGCTCTGCGACCGTTGGTGATCAAACAAGAACCCCGCGAAGAGGACGAAGCCAAACAGCT

GGCCCTCGCCGGCCTCTTTCCGTATCAACCTCCACCCCCACCTCCACCTTCTCACCCACACCCACACCCT

CCCCCAGCTCACCTGGCAGCTCCCCACTTGCAGTTCCAGATCGCGCACTGCGGACAGACAACCATGCACC

TCCAACCAGGTCACCCCACTCCACCCCCTACTCCAGTGCCCTCTCCTCACCCTGCACCTGCACTGGGTGC

CGCAGGATTGCCTGGCCCAGGCTCAGCACTGAAAGGCCTGGGTGCGGCTCACCCAGATCTTAGAGCCTCA

GGCGGAAGTGGAGCGGGCAAGGCCAAAAAGTCTGTGGACAAGAACAGCAACGAGTATCGGGTGCGGCGCG

AGCGCAATAACATTGCCGTCCGGAAGTCCCGCGATAAGGCCAAGCAGAGGAACGTGGAAACGCAGCAGAA

GTGCTGGAGCTGACCAGTGACAATGACCGCCTGAGGAAACGGGTCGAGCAGTTGAGCAGGGAACTGGATA

CCCTGCGTGGGATATTCCGGCAGCTTCCCGAGAGCTCCCTGGTCAAAGCGATGGGCAATTGCGCTTGAGG

St op

CTGAATCTAGA

St op *XbaI*

B

C/EBPα2–linker
AATTCAATGGAGTCAGCCGACTTTTACGAAGCCGAGC
gttacctcagtcggctgaaaatgcttcggctcggatc

C/EBPα3–linker
AATTCAATGAGCTCACACCTGCAATCTCCTCCCCACGCACCC
gttactcgagtgtggacgttagaggaggggtgcgtgggagct

C/EBPα4–linker
AATTCAATGCCAGGGGGTGC
gttacggtccccccacg

Supplementary figure 2. Generation of expression plasmids for human C/EBPα-variants. For a systematic analysis of the role(s) of C/EBPα-variants in the regulation of SIRT7-transcription plasmids for expression of the four known variants of human C/EBPα were generated. (**A**) A plasmid, pEX-A258-hCEBPA, containing a synthetic gene encoding

the longest human protein variant was used as starting material (Eurofins Genomics, Ebersberg, Germany). Within this codon-optimized synthetic gene restriction sites (red) were placed, which allowed excision of various 5´-fragments of the longest open reading frame. Alternative transcriptional start sites (TSS), which lead to the expression of C/EBPα_1, C/EBPα_2, C/EBPα_3 and C/EBPα_4, are numbered accordingly. The color-coding of the TSSs resulting in shortened proteins matches that used in (**B**). First, the EcoRI-XbaI-fragment from pEX-A258-hCEBPA was ligated into the analogously digested backbone of the eukaryotic expression vector pFLAG-CMV4 to generate pFLAG-CMV4-hCEBPA1. To generate shortened variants, pFLAG-CMV4-hCEBPA1 was double-digested using EcoRI and either AvrII or XhoI or FspAI as indicated. Then, matching double-stranded linker-oligonucleotides (**B**) were ligated into the respective linearized plasmids, resulting in plasmids expressing the three shorter variants of C/EBPα (pFLAG-CMV4-hCEBPA2-4).

Vector/plasmid name	Amount per transfection (ng)	Characteristic
pRL-SV40	50	Constitutive active Renilla luciferase vector without C/EBP binding-sites in SV40 promoter
pGL3-promoter	450	Empty firefly luciferase reporter-gene plasmid
pGL3-promoter SIRT7	450	Firefly luciferase reporter-gene plasmid with SIRT7 promoter-region containing C/EBP binding-sites 1 and 2
pFLAG-CMV4	1500	Empty pFLAG-CMV4 expression plasmid
pFLAG-CMV4 C/EBPα_1	1500	C/EBPα isoform 1 expression plasmid
pFLAG-CMV4 C/EBPα_2	1500	C/EBPα isoform 2 expression plasmid
pFLAG-CMV4 C/EBPα_3	1500	C/EBPα isoform 3 expression plasmid
pFLAG-CMV4 C/EBPα_4	1500	C/EBPα isoform 4 expression plasmid
pcDNA3.1/HisC	1500	Empty pcDNA3.1/HisC expression plasmid
pcDNA3.1/HisC C/EBPβ_1	1500	C/EBPβ isoform 1 (LAP1) expression plasmid[15]
pcDNA3.1/HisC C/EBPβ_2	1500	C/EBPβ isoform 2 (LAP2) expression plasmid[15]
pcDNA3.1/HisC C/EBPβ_3	1500	C/EBPβ isoform 3 (LIP) expression plasmid[15]
pCMV6-entry	1500	Empty pCMV6-entry expression plasmid
pCMV6-entry C/EBPε	1500	C/EBPε expression plasmid

Supplementary table 4. Vectors/plasmids for reporter-gene assays.

Western blot analysis

Whole cell lysates of AR230 and Ba/F3 cells were separated on 10% SDS-polyacrylamide gels. Proteins were transferred onto polyvinylidene fluoride (PVDF) membranes using semi-dry blotting at 0.8 mA/cm² for 30 min[16]. After blocking in WB-T buffer (10 mM Tris/HCl pH 7.5, 100 mM NaCl, 0.1% (v/v) Tween 20) with 5% (w/v) skimmed milk powder, the membrane was incubated overnight with primary antibodies as described in supplementary table 3. After washing in WB-T buffer and a repeated blocking in WB-T buffer with 5% (w/v) milk powder, the appropriate HRP-conjugated secondary antibodies were added (1:10.000 goat anti-rabbit; 1:5.000 goat anti-mouse, Santa Cruz biotechnology, Dallas, USA). Chemiluminescence-based detection of proteins was carried out with the LAS-500 Imaging system (GE Healthcare, Chicago, IL, USA).

Survival analysis

Transcriptomic datasets were extracted from the Gene Expression Omnibus (GEO) repository at the National Center for Biotechnology Information (NCBI) (http://www.ncbi.nlm.nih.gov/geo). We used the dataset by Metzeler[17] (GEO ID GSE12417, GPL96 subset, 163 samples). SIRT7 gene expression levels were assessed using the probe set ID 218797_s_at. FLT3-ITD mutated AML patients (n=81) and FLT3 wild-type AML patients (n=82) were grouped according to their expression level of SIRT7 into SIRT7 high and SIRT7 low expression. Cut-offs between the 10th and the 90th percentile of SIRT7-expression values, incremented in steps of 5, were tested to obtain the best separating cut-off value leading to 9.06. Survival analysis (overall survival) was performed by applying the Kaplan-Meier estimator from the Survival Analysis package[18] and the statistic software package R (www.r-project.org). To test for statistical significance, we performed a log-rank test.

Statistics

Statistical analyses and generation of figures were carried out using the SigmaPlot 13 software. Data are presented as means±SEM. Initially, normal distribution of values was tested using the Shapiro-Wilk method. Normally distributed values were compared to another group using Student´s t-test. In the case of non-normally distributed values two groups were compared by the Mann-Whitney Rank sum test, as indicated. For all tests, the significance criterion $p<0.05$ was used. All numbers of replications (n) in figure legends refer to biological replicates.

Supplement – Results

CML: SIRT7-expression and SIRT7 protein-levels are influenced by BCR-ABL inhibition

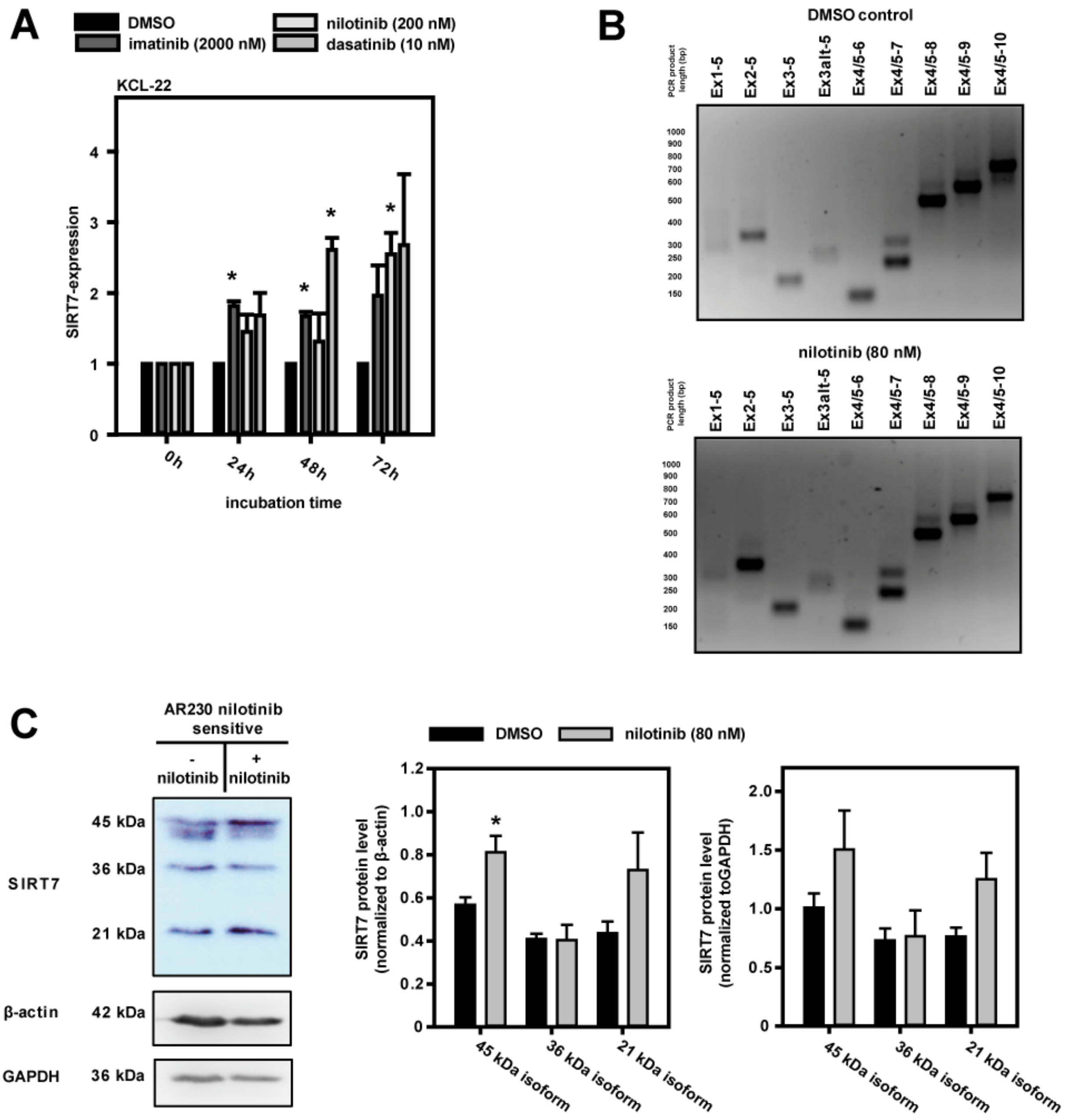

Supplementary figure 3. SIRT7 in CML.

(A) BCR-ABL in KCL-22 CML cells was inhibited by imatinib, nilotinib and dasatinib (negative control DMSO). SIRT7-expression was measured after 0, 24, 48 and 72 hours. The expression increase was associated on BCR-ABL inhibition by the different TKIs. Mean±SEM. value normalization to 0 h sample. n=3. Significant differences corresponding to DMSO control were identified with Student´s t-test (*p<0.05). **(B)** BCR-ABL in AR230

nilotinib-sensitive cells was inhibited by nilotinib. In exon-specific SIRT7 reverse transcription-PCR no qualitative changes in exon usage were detectable. Representative stained 2% (w/v) agarose gels are shown (n=3). **(C)** BCR-ABL in AR230 nilotinib-sensitive cells was inhibited for 24 h by nilotinib. Main SIRT7-isoforms (45 kDa, 36 kDa, 21 kDa) protein levels were analyzed in whole cell lysates by western blotting. The 45 kDa and 21 kDa isoforms protein levels increased after TKI treatment. Mean±SEM. Value normalization to house-keeping proteins β-actin or GAPDH. n=3. Significant differences corresponding to DMSO control were identified with Student´s t-test (*$p<0.05$).

AML: SIRT7-expression depends on FLT3-ITD mutation

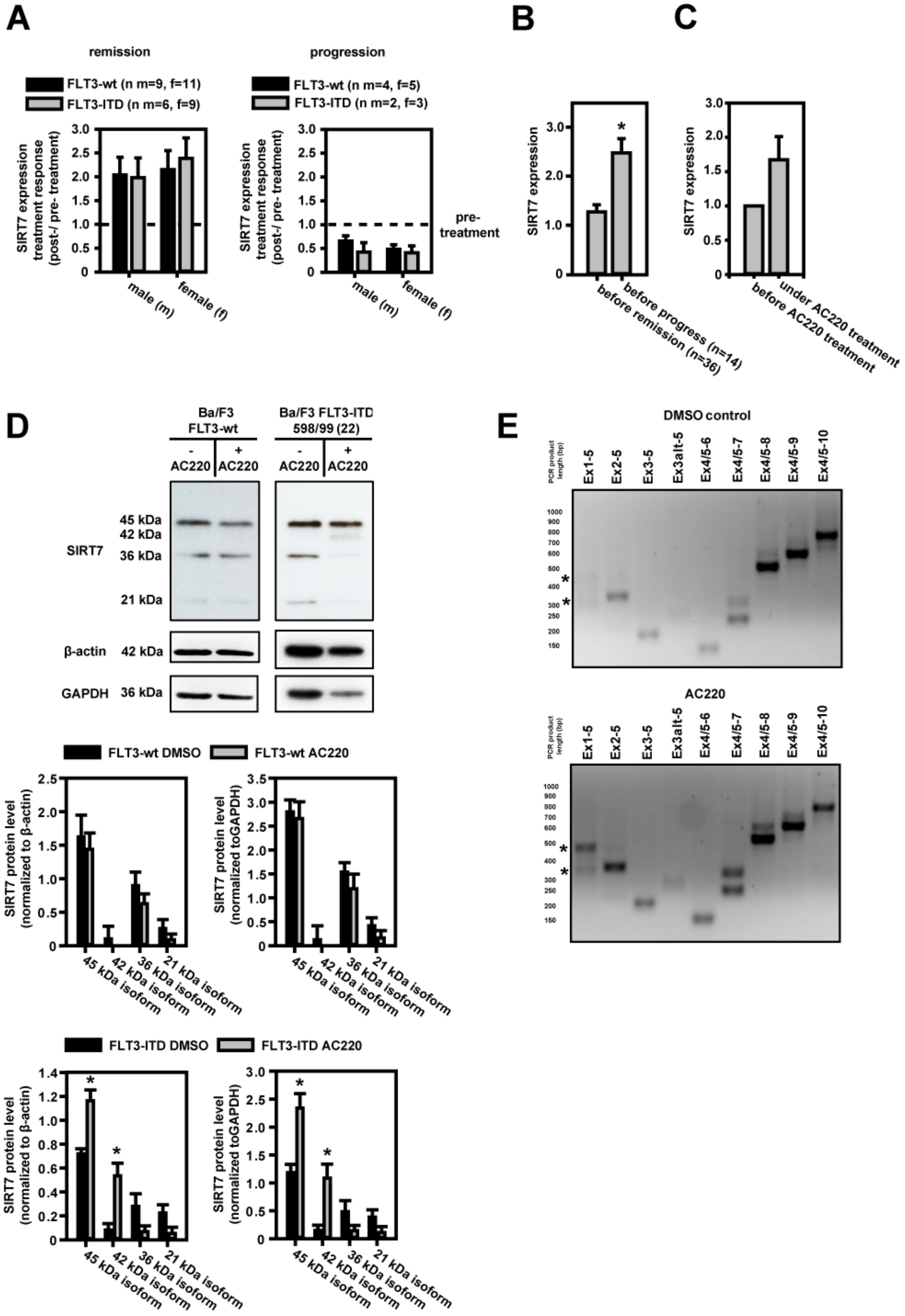

Supplementary figure 4. SIRT7 in AML.

(A) SIRT7-expression in AML patients bone marrow depended on treatment response. Identical characteristics were found in FLT3-wt and FLT3-ITD mutated leukemias (mean±SEM; Mann-Whitney Rank sum test (*p<0.05)). **(B)** SIRT7-expression in pre-treated AML patients bone marrow differs between treatment response (mean±SEM; Mann-Whitney Rank sum test (*p<0.05)). **(C)** SIRT7-expression in AML-patients increased in times of positive treatment effects by AC220 (n=3; mean±SEM; t-test (*p<0.05)). **(D)** FLT3-ITD in Ba/F3 FLT3-ITD 598/99(22) cells was inhibited for 24 h by AC220. Negative control was Ba/F3 FLT3-wt cells. SIRT7 protein-isoforms were analyzed in whole cell lysates by western blotting. The 45 kDa-isoform increased after FLT3-ITD inhibition. The 36 kDa- and 21 kDa-isoform levels were reduced. Furthermore, a new unknown 42 kDa-isoform was detected. Mean±SEM. Value normalization to housekeeper β-actin or GAPDH. n=3. Significant differences corresponding to DMSO control were identified with Student´s t-test (*p<0.05). **(E)** FLT3-ITD in MV4-11 cells was inhibited by AC220. In exon-specific SIRT7 reverse transcription-PCR, a qualitative change in exon usage was detectable. Upon AC220-treatment a new exon 1-5-containing PCR-product was detected (150 bp difference to exon 2-5-containing PCR-product, indicated by *). A representative stained 2% (w/v) agarose gel is shown (n=3).

AML: Low SIRT7-expression correlates with poor prognosis in FLT3-ITD mutated and FLT3 wild-type AML patients

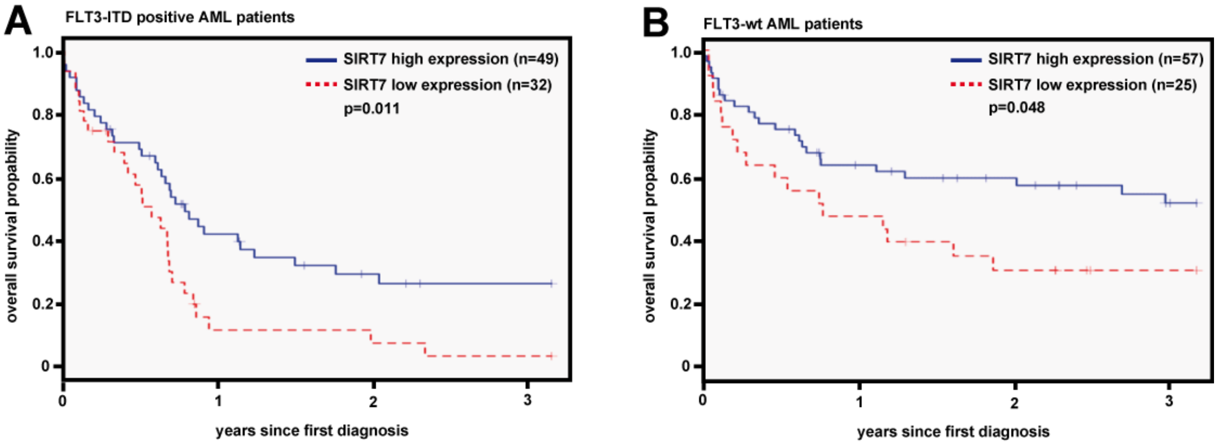

Supplementary figure 5. Survival analysis: Low SIRT7-expression correlated with poor prognosis in FLT3-ITD mutated and FLT3 wild-type AML patients.

Kaplan-Meier curves of FLT3-ITD-positive **(A)** and FLT3-wt **(B)** AML patients are shown. Overall survival probability of patients with high SIRT7-expression was significantly higher than in the low SIRT7-expression group. Significant changes were identified by log-rank test ($p<0.05$).

C/EBPs α, β and ε: missing links in FLT3-ITD/SIRT7, BCR-ABL/SIRT7 and monocyte differentiation/SIRT7 relationship

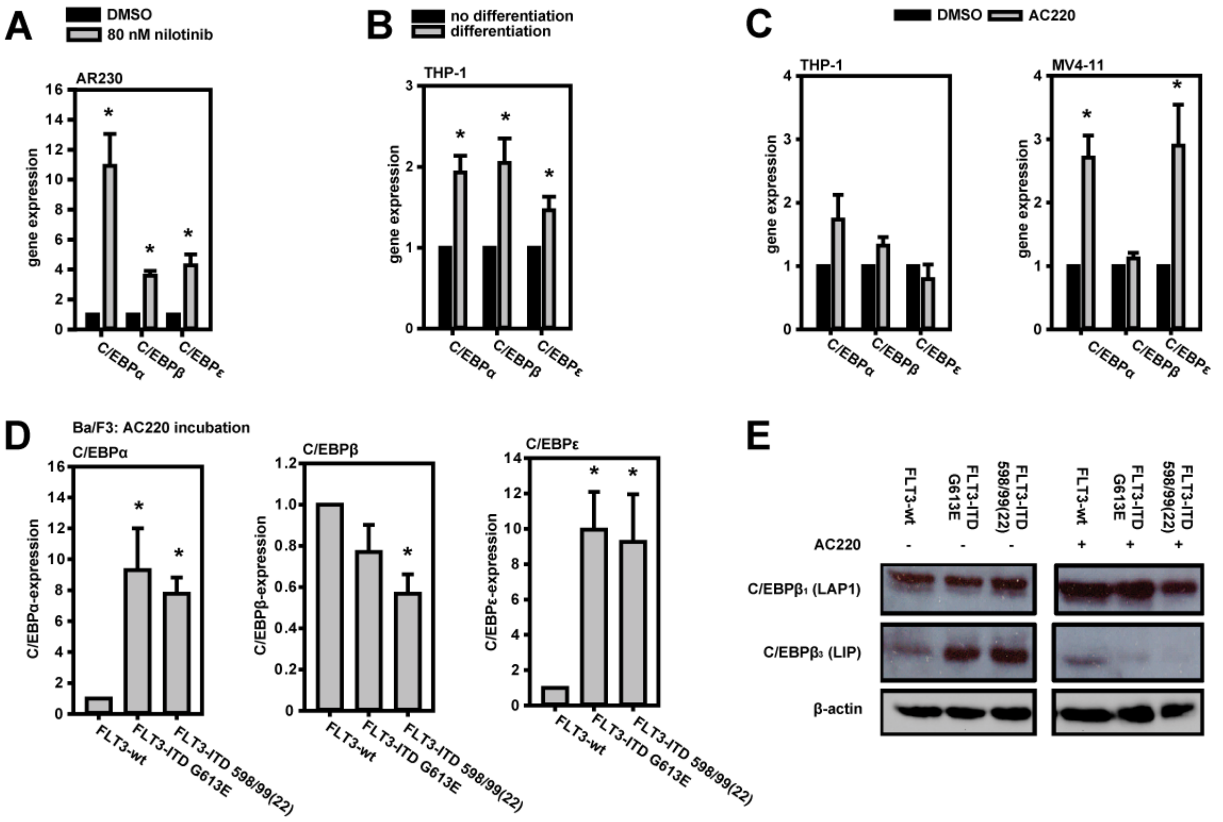

Supplementary figure 6. C/EBPα, -β and –ε, the missing links in FLT3-ITD/SIRT7, BCR-ABL/SIRT7 and monocyte differentiation/SIRT7 relationship.

Inhibition- or differentiation-dependent changes of C/EBPα, -β and -ε-expression were measured in different disease-specific cell lines by qRT-PCR. **(A)** BCR-ABL inhibition by nilotinib in AR230 nilotinib-sensitive cells or **(B)** PMA-induced THP-1 monocyte differentiation resulted in increased C/EBPα, -β and -ε-expression. **(C+D)** C/EBPα and -ε-expression increased after FLT3-ITD inhibition by AC220 in MV4-11 and Ba/F3 FLT3-ITD cells. C/EBPβ-expression was unaffected (MV4-11 cells) or reduced (Ba/F3 cells). Mean±SEM. n=3. Significant differences corresponding to no differentiation (B) or inhibitor treatment (A, C, D) were identified with Student´s t-test (*p<0.05). **(E)** FLT3-ITD in Ba/F3 FLT3-ITD G613E and FLT3-ITD 598/99(22) cells was inhibited by treatment with AC220 for 24 h. Negative controls were Ba/F3 FLT3-wt cells. C/EBPβ protein levels were analyzed in whole cell lysates by

western blotting. C/EBPβ_1 (LAP1) isoform (activating isoform) changes were not detectable after AC220 treatment. C/EBPβ_3 (LIP) isoform (inhibitory isoform) protein levels decreased FLT3-ITD-dependently after AC220 treatment (previously described by Haas et al[19]). n=1.

Supplement - References

1. Muller MC, Erben P, Saglio G, Gottardi E, Nyvold CG, Schenk T, et al. Harmonization of BCR-ABL mRNA quantification using a uniform multifunctional control plasmid in 37 international laboratories. Leukemia 2008 Jan; 22(1): 96-102.

2. Ni N, Zhang D, Xie Q, Chen J, Wang Z, Deng Y, et al. Effects of let-7b and TLX on the proliferation and differentiation of retinal progenitor cells in vitro. Scientific reports 2014 Oct 20; 4: 6671.

3. Mohrin M, Shin J, Liu Y, Brown K, Luo H, Xi Y, et al. A mitochondrial UPR-mediated metabolic checkpoint regulates hematopoietic stem cell aging. Science 2015; 347(6228): 1374-1377.

4. Zhang J, Li L, Baldwin AS, Jr., Friedman AD, Paz-Priel I. Loss of IKKbeta but Not NF-kappaB p65 Skews Differentiation towards Myeloid over Erythroid Commitment and Increases Myeloid Progenitor Self-Renewal and Functional Long-Term Hematopoietic Stem Cells. PLoS One 2015; 10(6): e0130441.

5. Zhang L, Friedman AD. SHP2 tyrosine phosphatase stimulates CEBPA gene expression to mediate cytokine-dependent granulopoiesis. Blood 2011 Aug 25; 118(8): 2266-2274.

6. Valiuliene G, Stirblyte I, Jasnauskaite M, Borutinskaite V, Navakauskiene R. Anti-leukemic effects of HDACi Belinostat and HMTi 3-Deazaneplanocin A on human acute promyelocytic leukemia cells. Eur. J.Pharmacol. 2017 Mar 15; 799: 143-153.

7. Malu K, Garhwal R, Pelletier MG, Gotur D, Halene S, Zwerger M, et al. Cooperative Activity of GABP with PU.1 or C/EBPepsilon Regulates Lamin B Receptor Gene Expression, Implicating Their Roles in Granulocyte Nuclear Maturation. J Immunol. 2016 Aug 1; 197(3): 910-922.

8. Pfaffl MW. A new mathematical model for relative quantification in real-time RT-PCR. Nucleic Acids Res. 2001 May 1; 29(9): e45.

9. Mahon FX, Deininger MW, Schultheis B, Chabrol J, Reiffers J, Goldman JM, et al. Selection and characterization of BCR-ABL positive cell lines with differential sensitivity to the tyrosine kinase inhibitor STI571: diverse mechanisms of resistance. Blood 2000 Aug 1; 96(3): 1070-1079.

10. Grundler R, Thiede C, Miething C, Steudel C, Peschel C, Duyster J. Sensitivity toward tyrosine kinase inhibitors varies between different activating mutations of the FLT3 receptor. Blood 2003 Jul 15; 102(2): 646-651.

11. Arreba-Tutusaus P, Mack TS, Bullinger L, Schnoder TM, Polanetzki A, Weinert S, et al. Impact of FLT3-ITD location on sensitivity to TKI-therapy in vitro and in vivo. Leukemia 2016 May; 30(5): 1220-1225.

12. Ford E, Voit R, Liszt G, Magin C, Grummt I, Guarente L. Mammalian Sir2 homolog SIRT7 is an activator of RNA polymerase I transcription. Genes & development 2006 May 1; 20(9): 1075-1080.

13. Shin J, He M, Liu Y, Paredes S, Villanova L, Brown K, et al. SIRT7 represses Myc activity to suppress ER stress and prevent fatty liver disease. Cell Rep 2013 Nov 14; 5(3): 654-665.

14. Steinberg XP, Hepp MI, Fernandez Garcia Y, Suganuma T, Swanson SK, Washburn M, et al. Human CCAAT/enhancer-binding protein beta interacts with chromatin remodeling complexes of the imitation switch subfamily. Biochemistry 2012 Feb 7; 51(5): 952-962.

15. Henriquez B, Hepp M, Merino P, Sepulveda H, van Wijnen AJ, Lian JB, et al. C/EBPbeta binds the P1 promoter of the Runx2 gene and up-regulates Runx2 transcription in osteoblastic cells. J Cell Physiol. 2011 Nov; 226(11): 3043-3052.

16. Kyhse-Andersen J. Electroblotting of multiple gels: a simple apparatus without buffer tank for rapid transfer of proteins from polyacrylamide to nitrocellulose. J Biochem Biophys Methods 1984 Dec; 10(3-4): 203-209.

17. Metzeler KH, Hummel M, Bloomfield CD, Spiekermann K, Braess J, Sauerland MC, et al. An 86-probe-set gene-expression signature predicts survival in cytogenetically normal acute myeloid leukemia. Blood 2008 Nov 15; 112(10): 4193-4201.

18. Therneau TM. A Package for Survival Analysis in S. 2015.

19. Haas SC, Huber R, Gutsch R, Kandemir JD, Cappello C, Krauter J, et al. ITD- and FL-induced FLT3 signal transduction leads to increased C/EBPβ-LIP expression and LIP/LAP ratio by different signalling modules. Br. J. Haematol. 2010; 148(5): 777-790.
